# Use of anchoring vignettes to evaluate health reporting behavior amongst adults aged 50 years and above in Africa and Asia – testing assumptions

**DOI:** 10.3402/gha.v6i0.21064

**Published:** 2013-09-05

**Authors:** Siddhivinayak Hirve, Xavier Gómez-Olivé, Samuel Oti, Cornelius Debpuur, Sanjay Juvekar, Stephen Tollman, Yulia Blomstedt, Stig Wall, Nawi Ng

**Affiliations:** 1 Vadu Rural Health Program, KEM Hospital Research Centre, Pune, India; 2 Division of Epidemiology and Global Health, Department of Public Health and Clinical Medicine, Umeå Centre for Global Health Research, Umeå University, Umeå, Sweden; 3 School of Public Health, MRC/Wits Rural Public Health and Health Transitions Research Unit, University of the Witwatersrand, Johannesburg, South Africa; 4 African Population and Health Research Center, Nairobi, Kenya; 5 Navrongo Health Research Center, Navrongo, Ghana

**Keywords:** reporting heterogeneity, mobility, cognition, self-rating, anchoring vignettes, vignette equivalence, response consistency

## Abstract

**Background:**

Comparing self-rating health responses across individuals and cultures is misleading due to different reporting behaviors. Anchoring vignettes is a technique that allows identifying and adjusting self-rating responses for reporting heterogeneity (RH).

**Objective:**

This article aims to test two crucial assumptions of vignette equivalence (VE) and response consistency (RC) that are required to be met before vignettes can be used to adjust self-rating responses for RH.

**Design:**

We used self-ratings, vignettes, and objective measures covering domains of mobility and cognition from the WHO study on global AGEing and adult health, administered to older adults aged 50 years and above from eight low- and middle-income countries in Africa and Asia. For VE, we specified a hierarchical ordered probit (HOPIT) model to test for equality of perceived vignette locations. For RC, we tested for equality of thresholds that are used to rate vignettes with thresholds derived from objective measures and used to rate their own health function.

**Results:**

There was evidence of RH in self-rating responses for difficulty in mobility and cognition. Assumptions of VE and RC between countries were violated driven by age, sex, and education. However, within a country context, assumption of VE was met in some countries (mainly in Africa, except Tanzania) and violated in others (mainly in Asia, except India).

**Conclusion:**

We conclude that violation of assumptions of RC and VE precluded the use of anchoring vignettes to adjust self-rated responses for RH across countries in Asia and Africa.

The debate on measurement issues in social science over the last few decades has been mainly on advanced methodologies such as path analysis and structural equation modeling to address concerns of data collection, measurement error, and ordinal data (1–3). Somehow, the more serious concern of lack of interpersonal comparability in survey responses has largely been ignored by social scientists (4). Even the debate over ‘ordinal’ scale versus ‘interval’ scale finds scarce reference to the problem of interpersonal incomparability (5). Surveys often use rank categories or self-ratings to measure traits of interest. With rank categorizations, measures are placed in ordered categories. With self-ratings, respondents are asked to rate, for example, their health on an increasing Likert scale from ‘poor’ to ‘excellent’ health. Such ordered ordinal responses are analyzed with the assumption of an underlying latent interval scale. For such analyses, the tendency is to treat one person's categorization or rating response to be the same as that of another person and assume that both understand the response categories in the same way. In other words, we assume that individual's self-rate their response using the same cut-off points or thresholds on the latent interval scale which differentiate the categories ‘poor’, ‘fair’, ‘good’, or ‘excellent’ on the manifest scale. However, there is a large body of evidence to suggest that individuals or groups of individuals interpret and choose categories in vastly different ways. Two individuals or groups of individuals with identical health levels may rate their own health differently based on their understanding, experience, and expectation of their own health (6). This difference in reporting style or reporting behavior is referred to as response-category differential item functioning (7) or reporting heterogeneity (RH) (8). RH has been seen across sexes (9), socio-economic strata (10), race and ethnicities (11, 12), and countries (13–16). Unless recognized, such RH can result in misleading and incorrect interpretations (7, 17).

In recent years, ‘anchoring vignettes’ has been shown to be a promising strategy to overcome the problem of RH in survey questions (14, 18). Anchoring vignettes are brief texts describing a hypothetical character who exemplifies a certain fixed level of the trait of interest. The respondent is asked to rate the level of the trait for the vignette character as she/he would do for his/her own. The vignette ratings are used to identify the problem of RH and then adjust the self-rating response by removing its systematic variation using either a parametric or non-parametric approach (8, 18–20). ‘Anchoring vignettes’ method has increasingly been used to improve interpersonal and cross-cultural comparability of survey questions in areas of political efficacy, work disability, job satisfaction, life satisfaction, health and health system responsiveness (7, 8, 21–26).

The anchoring vignettes approach requires two fundamental assumptions to be met – vignette equivalence (VE), that is, all respondents understand the health state described by a vignette in the same way; and response consistency (RC), that is, a respondent uses the same thresholds to rate vignettes as she/he does to rate his/her own self. The VE assumption allows for the identification of RH, if any, while the assumption of RC is necessary for adjusting self-rating responses for RH. Violation of either assumption precludes the use of anchoring vignettes to correct self-rating responses for RH. Initial studies have used informal checks to assess inconsistencies in rank ordering of vignette severity or less stringent non-parametric methods such as testing for systematic difference in vignette rankings to evaluate these assumptions (19, 26). Analytic methods are now developed to allow a more rigorous evaluation of measurement assumptions using parametric methods (20, 27–29).

The World Health Organization (WHO) study on global AGEing and adult health (SAGE) conducted at eight surveillance sites of the International Network for the Demographic Evaluations of Populations and their Health (INDEPTH) Network aims to compile comprehensive longitudinal data on the health and well-being of older adult and elderly populations across different low- and middle-income countries (30). In this article, we use the SAGE data on self-ratings and vignettes in mobility and cognition to test the assumptions of VE and RC that are essential for the use of the anchoring vignettes approach.

## Methods

### Ethics statement

The Ethics Review Committee of the WHO, Geneva and respective Ethics Committees of the participating Health and Demographic Surveillance System (HDSS) sites of the INDEPTH Network approved the WHO SAGE. All respondents participated in the study after having completed an informed written consent.

### SAGE data

SAGE has adapted and built further on to the methods and instruments developed by the WHO for the World Health Survey that was conducted in 2002–03 in 70 countries. The SAGE questionnaire was pre-tested in 2005 amongst 1,500 respondents in India, Ghana, and Tanzania. The WHO's collaboration with the INDEPTH Network supported eight HDSS sites in Africa (Navrongo, Ghana; Nairobi, Kenya; Agincourt, South Africa; Ifakara, Tanzania) and Asia (Matlab, Bangladesh; Vadu, India; Purworejo, Indonesia; Filabavi, Vietnam) to implement an adapted summary version of SAGE (31). Three of these sites (Navrongo, Agincourt, and Vadu) also implemented the full version of SAGE in a smaller subset of its population. All sites represent predominantly rural populations except the urban slum site of Nairobi, Kenya. The cognitive ability of respondents to understand terms and concepts such as self-rating and vignette rating was ascertained at the start of the interview. Show cards were provided to aid respondents in their rating responses on the five-point Likert scale. Proxy respondents who knew the respondent well enough were identified and interviewed on behalf of the respondents with impaired ability to respond. A subset of respondents was re-tested for data quality assurance.

The summary version of SAGE included two self-rating questions on difficulty in functional ability in each of the eight domains (mobility, cognition, affect, self-care, vision, pain, sleep, and interpersonal relationships). These data were enhanced by linking with socio-demographic characteristics (age, sex, marital status, socioeconomic status (SES), family size, etc.) from each of the HDSS.

### Vignettes data

The vignettes were administered as part of the summary version of SAGE by all sites, except for Navrongo and Agincourt, which administered vignettes only as part of the fuller version of SAGE. Each domain included two self-rating questions (one for a lower and another for a higher level of functional ability) followed by five vignettes adapted from the WHO World Health Survey describing varying levels of severity of limitation of function (Appendix 1). The names of the hypothetical persons in the vignettes were chosen to be related to the same sex as the respondent and culturally appropriate. Respondents were advised to think of the hypothetical person's experience in the vignette as if they were their own. The vignette rating questions were identical to the two self-rating questions replacing ‘self’ with the name of the hypothetical person in the vignette. Vignettes were paired into four domain sets (mobility and affect; pain and relationships; sleep and vision; and care and cognition). The selected respondents were randomly allocated to four groups and one set of paired domain vignettes was administered to each group. The vignettes in a set were administered in no particular order of domain or severity. Respondents assessed the functional ability of their own self and that of the hypothetical persons in the vignettes, on a five-point ordinal scale of increasing difficulty (no difficulty, mild, moderate, severe, and extreme difficulty).

### Objective health measures

The fuller version of SAGE, in addition to the summary version, included some objective measures. Mobility was assessed by the time taken to walk four meters at normal and rapid speed. Handgrip strength (kg) was measured separately for both hands using Smedley's hand dynamometer. Cognition measures included immediate and delayed word recall, forward and backward digit span test, and verbal fluency. The average of the number of correct words recalled (where sequence did not matter) from a list of 10 words from 3 trials was taken as the score for the word recall test (maximum possible score 10). The length of the longest series of digits recalled by a respondent in the correct sequence was taken as the score for the forward and backward digit span test (maximum possible score 9). The number of animals listed by the respondent in 1 minute was taken as the score for the verbal fluency test. Each cognition test measure was rescaled from 0 to 1, with the higher score indicating higher cognition.

Sites implemented the summary version of SAGE either amongst *all* eligible older adults aged 50 years and above or on a random sample. Furthermore, the fuller version of SAGE was implemented in a smaller random subset of 500 adults aged 50 years and above at the Navrongo, Agincourt, and Vadu sites. For this article, we focus our analysis on the two self-ratings of mobility (difficulty in moving around, difficulty in performing vigorous activity) and cognition (difficulty in memory, difficulty in learning) as objective measures needed to test assumptions of vignettes were available for these domains.

### Statistical methods – testing assumptions

Consistency of orderings of the five vignettes was checked using the ‘ANCHORS’ package in R statistical programming language (32). Hierarchical ordered probit (HOPIT) models for testing VE and RC assumptions were developed in STATA. The VE test tested that there was no systematic variation in the perceived difference in the states described by any two vignettes. This was based on the observation that the perceived location (on the latent scale) of vignettes would be constant if VE held. We specified a HOPIT model for *V* ^*^_ij_, the perceived location of vignette *j* by respondent *i*. To achieve model identification, we constrained the location of vignette severity level 5 to zero and estimated the locations of the other vignettes relative to the reference vignette. We included interaction terms between each vignette and covariate (e.g. between first vignette and age groups) and tested for *all* parameters of the vignette–covariate interactions (Wald's test) to be equal to zero (global test for VE) (27). We also tested for individual covariate and vignettes interaction parameters to be equal to zero to determine which covariates influenced VE. We also assessed VE by a visual comparison across sites of the predicted locations of the vignettes stratified by site.

Testing for RC required information on objective measures in addition to vignettes data. Such objective measures were presumed to capture all the co-variation between the latent construct of interest and the observable characteristics that may influence RH. If so, then any systematic variation that was seen in self-assessment that remained after conditioning on these objective measures could be attributed to RH. We were only able to test for RC in Navrongo, Agincourt, and Vadu as objective measures needed to test the assumption were only available for these three sites. To test the assumption of RC, we compared the locations of response category thresholds estimated from vignette ratings with the threshold locations estimated from objective measures. To do this, we specified three HOPIT models – model 1 specified the perceived location of the vignette; model 2 specified the perceived location of the latent self-rating from all objective measures; model 3 was a special case of model 1 (vignettes) and 2 (objective measures) combined where the response category thresholds were identical. We then used likelihood ratio (LR) test to determine if model 3 was significantly different from models 1 and 2 together, for all covariates (global test for RC) and for each individual covariate to determine which covariate influenced RC violation. We also assessed RC across sites by a visual comparison of the thresholds predicted by the vignettes model and those predicted by the objective measure model.

For all HOPIT models, we normalized the location parameters by excluding the intercept and also allowed response category thresholds to vary by sex, age, and education (27). All model parameters were estimated by maximum likelihood.

## Results

The eight sites together had an estimated population of 107,900 individuals aged 50 years and above under demographic surveillance. Of the 38,793 individuals who participated in SAGE, 36,170 (93%) were administered vignettes in the different domains – 9,375 for mobility and affect; 8,788 for self-care and cognition; 9,205 for pain and relationships; 8,802 for vision and sleep. The Kenya site administered vignettes to a random sample of 781 out of 1,991 respondents, whereas vignettes could not be administered to 29 respondents in the Indonesia site. Self-rating responses were missing for less than 1%. About 4 and 7% of respondents were not administered the timed walk and the grip strength test, respectively, while the cognitive tests could not be administered in less than 1% of the respondents. The VE assumption was tested on 9,375 and 8,788 individuals who responded to the mobility and cognition vignettes, respectively. The RC assumption was tested on the subset of 293 and 373 individuals who were administered the objective measures of mobility and cognition, respectively.

Table 1 describes the socio-demographic profile of the participants across the sites. The overall mean age of men was 63.5 years and that of women was 64.1 years. Participants from Kenya, Tanzania, Bangladesh, and Vietnam were significantly younger when compared to those from Ghana, while there was no significant difference in age between participants from South Africa, India, Indonesia, and Ghana. Overall, 47% of participants were men (range: 32% in South Africa to 65% in Kenya). Overall, 39% of participants had none or less than primary education; more than 90% in Ghana, South Africa, and India and only 10% in Vietnam. Overall, 13% of participants (about 11% in African sites, about 4% in India and Indonesia, and 25 and 29% in Vietnam and Bangladesh, respectively) rated their own health as bad or very bad. There were no clear patterns in self-ratings for difficulty in functional ability in any of the domains across sites though it appeared that overall Bangladesh reported higher difficulties compared with other sites. The Asian sites (except Bangladesh) reported significantly lower difficulty in moving around compared to the African sites. This pattern was less apparent for self-ratings for difficulty with vigorous activity. Similarly, it also appeared that Bangladesh reported higher difficulty with memory compared to other Asian and African sites. This pattern however was less apparent for self-ratings for difficulty with learning. Based on objective measures, participants from South Africa were significantly less agile (normal walk speed) compared to Ghana and India. However, there was no significant difference in mobility across the three sites as measured by rapid walk speed (Table 1). Participants from Ghana were significantly stronger (grip strength) compared to South Africa and India. Participants from Ghana had significantly better scores (immediate verbal recall test) compared to South Africa and India. There was no significant difference in scores across sites for all other cognition tests (except significantly lower scores on verbal fluency for participants from South Africa compared to Ghana).

**Table 1:** Socio-demographic and health characteristics (means and proportions; SD in parenthesis) of men and women (*N*=37,409)

	Navrongo, Ghana (*n*=597)	Nairobi, Kenya (*n*=1,991)	Agincourt, South Africa (*n*=438)	Ifakara, Tanzania (*n*=5,024)	Matlab, Bangladesh (*n*=4,004)	Vadu, India (*n*=5,086)	Purworejo, Indonesia (*n*=11,753)	Filabavi, Vietnam (*n*=8,516)
Mean age in years (SD)	64.3 (9.91)	59.0 (8.89)	65.2 (10.6)	62.5 (9.2)	62.1 (8.98)	65.0 (9.26)	64.1 (9.41)	65.3 (10.7)
Males (%)	40	65	32	48	50	52	46	41
Education								
Primary or less (%)	96	28	91	39	56	91	29	10
Secondary (%)	2	57	4	56	29	6	55	49
Higher secondary/more (%)	2	15	5	4	15	3	16	41
No spousal support (%)	50	46	54	34	24	26	29	32
SES								
First quintile (poorest) (%)	28	24	16	19	15	11	20	14
Second quintile (%)	25	15	19	20	17	15	20	18
Third quintile (%)	22	20	19	21	18	22	20	21
Fourth quintile (%)	19	21	21	40	23	21	20	23
Fifth quintile (highest) (%)	6	19	25	0	27	31	19	23
SRH								
Very good (%)	3	11	7	4	2	3	2	0
Good (%)	40	50	42	52	28	53	66	15
Moderate (%)	43	26	38	34	40	40	29	60
Bad (%)	13	12	12	10	25	4	3	24
Very bad (%)	1	1	0	1	4	0	0	1
Mean self-rating^1^ for difficulty with								
Moving around	1.9 (1.06)	1.9 (1.06)	1.9 (1.17)	1.8 (1.08)	2.1 (1.23)	1.8 (0.86)	1.3 (0.68)	1.5 (0.94)
Vigorous activity	2.8 (1.28)	2.2 (1.21)	2.3 (1.37)	2.5 (1.29)	4.1 (1.11)	2.3 (1.04)	2.1 (1.21)	3.1 (1.42)
Sadness	1.4 (0.76)	1.5 (0.78)	1.2 (0.71)	1.2 (0.60)	1.7 (1.11)	1.6 (0.83)	1.2 (0.51)	1.2 (0.65)
Worry	1.5 (0.79)	1.5 (0.84)	1.2 (0.69)	1.3 (0.71)	1.6 (0.99)	1.7 (0.78)	1.2 (0.53)	1.2 (0.65)
Body aches	2.5 (0.98)	2.1 (1.02)	2.3 (1.20)	2.2 (1.08)	3.3 (1.19)	2.0 (0.85)	2.0 (0.85)	2.5 (1.15)
Discomfort	2.5 (0.96)	2.1 (1.07)	2.2 (1.16)	2.1 (1.04)	2.8 (1.02)	2.1 (0.88)	2.0 (0.85)	2.4 (1.17)
Relationships	1.9 (0.85)	1.8 (1.03)	2.0 (1.09)	1.8 (1.00)	2.5 (1.25)	1.7 (0.73)	1.8 (0.84)	2.1 (1.14)
Conflicts	2.1 (1.05)	1.8 (1.01)	2.2 (1.43)	1.8 (1.13)	2.3 (1.34)	1.9 (0.85)	2.0 (1.00)	2.3 (1.29)
Waking up	2.0 (1.12)	1.4 (0.73)	1.8 (1.29)	1.6 (1.03)	1.5 (0.94)	1.8 (0.75	1.3 (0.69)	1.7 (1.11)
Feeling rested	2.0 (1.05)	1.4 (0.76)	1.5 (0.99)	1.4 (0.93)	1.8 (1.14)	1.9 (0.79)	1.3 (0.66)	1.5 (0.98)
Far vision	2.1 (1.04)	1.8 (0.94)	2.5 (1.22)	1.8 (1.09)	2.7 (1.35)	1.8 (0.90)	1.8 (0.89)	2.6 (1.20)
Near vision	2.2 (0.97)	1.9 (1.01)	2.2 (1.20)	1.8 (1.03)	2.6 (1.19)	1.8 (0.85)	1.7 (0.89)	2.2 (1.14)
Bathing	2.1 (0.84)	1.8 (0.88)	2.2 (1.18)	1.8 (1.05)	2.8 (1.16)	1.9 (0.79)	1.4 (0.65)	1.7 (1.08)
Maintaining appearance	2.1 (0.96)	1.7 (0.87)	2.5 (1.25)	1.7 (0.98)	3.3 (1.27)	2.0 (0.87)	1.4 (0.66)	1.9 (1.13)
Memory	1.9 (1.23)	1.7 (0.88)	1.8 (1.06)	1.9 (1.14)	2.4 (1.34)	2.0 (0.87)	1.8 (0.95)	1.8 (1.19)
Learning	1.5 (0.99)	2.1 (1.15)	1.8 (1.06)	1.6 (1.03)	2.0 (1.08)	2.0 (0.88)	2.0 (1.02)	1.4 (0.86)
Mobility test measures^2^								
Normal four-meter walk	5.3 (8.5)	NA	6.5 (3.3)	NA	NA	4.7 (2.4)	NA	NA
Rapid four-meter walk	4.0 (8.3)		4.1 (2.3)			3.3 (1.78)		
Grip strength (right)	29.9 (9.9)		23.8(10.7)			19.9 (10.1)		
Grip strength (left)	28.7 (10.2)		23.2(10.2)			19.2 (9.3)		
Cognition test measures^2^								
Immediate verbal recall	.70 (.897)	NA	.54 (.186)	NA	NA	.47 (.128)	NA	NA
Delayed verbal recall	.61 (.859)		.46 (.241)			.39 (.185)		
Digit span (forward)	.49 (1.03)		.46 (.186)			.45 (.163)		
Digit span (backward)	.29 (1.19)		.19 (.182)			.28 (.165)		
Verbal fluency	.40 (.172)		.31 (.135)			.41 (.147)		

1 Self-ratings unadjusted for DIF. Ratings range from 1 = no difficulty to 5 = extreme difficulty.

2 Objective measures are rescaled on an improving scale of 0 to 1.

Overall, participants rated vignettes consistent with their order of severity in the mobility domain across all sites (Appendix 2). Similarly, there were no instances of incorrect ordering of vignettes in the cognition domain across all sites except in Kenya where learning vignette severity level 4 was incorrectly rated lower than vignette severity level 3 and in India where both memory and learning vignette severity level 5 was rated lower than vignette severity level 4 (Table 2). The proportion of ties between vignette pairs (especially for cognition vignette pair 4 and 5) was higher amongst Asian sites compared to Africa. However, there appeared to be no clear pattern of high proportion of ties between vignette pairs across sites either for mobility or for cognition.

**Table 2:** Mean ratings of vignettes for mobility (*N*=9,375) and cognition (*N*=8,788)

	Navrongo, Ghana	Nairobi, Kenya	Agincourt, S Africa	Ifakara, Tanzania	Matlab, Bangladesh	Vadu, India	Purworejo Indonesia	Filabavi Vietnam
Mobility – moving around								
	*n*=148	*n*=213	*n*=105	*n*=1,412	*n*=1,003	*n*=1,307	*n*=2,970	*n*=2,217
Vig1	1.27 (.792)	1.17 (.577)	1.44 (1.04)	1.30 (.711)	1.16 (.562)	1.55 (.855)	1.31 (.678)	1.13 (.509)
Vig2	2.26 (1.02)	1.95 (.982)	2.13 (1.17)	2.11 (1.02)	2.35 (1.17)	2.23 (.831)	2.01 (.929)	1.82 (1.03)
Vig3	3.59 (.730)	2.92 (1.03)	3.55 (1.16)	3.16 (1.09)	3.24 (1.01)	2.88 (.963)	2.61 (1.01)	3.01 (1.11)
Vig4	3.97 (.559)	3.83 (.622)	3.84 (.814)	3.62 (.916)	4.12 (.813)	3.44 (.956)	3.33 (.908)	3.79 (.779)
Vig5	4.72 (.750)	4.56 (.864)	4.5 (.928)	4.48 (.782)	4.67 (.561)	4.18 (1.18)	4.35 (.691)	4.82 (.497)
Mobility – vigorous activity								
Vig1	1.33 (.762)	1.22 (.632)	1.42 (.945)	1.37 (.775)	1.29 (.717)	1.70 (.939)	1.39 (.758)	1.29 (.727)
Vig2	2.93 (.99)	3.62 (1.09)	2.3 (1.23)	2.66 (1.08)	3.6 (1.13)	2.55 (.977)	2.55 (.975)	3.5 (.994)
Vig3	4.08 (.753)	3.96 (.953)	3.66 (1.08)	3.52 (.994)	4.19 (.943)	3.08 (.954)	3.17 (.962)	4.24 (.80)
Vig4	4.22 (.658)	4.31 (.80)	4.00 (.863)	3.72 (.913)	4.57 (.733)	3.53 (.944)	3.76 (.766)	4.47 (.679)
Vig5	4.75 (.768)	4.68 (.837)	4.56 (.882)	4.50 (.797)	4.83 (.480)	4.12 (.121)	4.48 (.674)	4.88 (.444)
Cognition – memory								
	*n*=151	*n*=186	*n*=110	*n*=1,152	*n*=1,001	*n*=1,251	*n*=2,890	*n*=2,047
Vig1	1.25 (.57)	1.37 (.695)	1.72 (1.21)	1.75 (.917)	1.13 (.493)	1.83 (.922)	1.31 (.668)	1.18 (.554)
Vig2	1.90 (.772)	2.03 (.77)	2.46 (1.03)	2.27 (.834)	2.01 (.797)	2.16 (.818)	1.87 (.80)	1.88 (.811)
Vig3	2.94 (.821)	3.06 (.80)	3.08 (.958)	2.6 (.932)	3.41 (.939)	3.24 (.859)	2.68 (.813)	3.26 (.852)
Vig4	3.49 (.721)	3.34 (.888)	3.40 (1.01)	3.06 (1.14)	3.99 (.822)	3.48 (.881)	3.33 (.767)	3.95 (.742)
Vig5	4.75 (.637)	4.25 (.637)	4.27 (1.15)	3.56 (1.41)	4.38 (.70)	3.33 (.949)	3.93 (.689)	4.36 (.744)
Cognition – learning								
Vig1	1.17 (.46)	1.40 (.78)	1.8 (1.29)	1.78 (.897)	1.20 (.584)	1.76 (.944)	1.35 (.708)	1.20 (.585)
Vig2	1.91 (.757)	1.73 (.943)	2.47 (1.08)	2.25 (.881)	1.85 (.928)	2.11 (.911)	1.82 (.856)	1.81 (.893)
Vig3	3.09 (.763)	3.28 (.874)	2.90 (1.06)	2.66 (.935)	3.48 (1.08)	3.35 (.793)	2.86 (.80)	3.56 (.804)
Vig4	3.51 (.775)	3.00 (1.14)	3.39 (1.08)	3.04 (1.13)	3.88 (1.0)	3.47 (.96)	3.44 (.776)	4.10 (.815)
Vig5	4.75 (.615)	4.38 (.705)	4.09 (1.18)	3.54 (1.39)	4.39 (.779)	3.38 (1.09)	4.02 (.707)	4.51 (.734)

Note: Vignettes 1–5 are ordered by increasing level of severity, vignette ratings range from 1 = none to 5 = extreme difficulty. SD in parenthesis.

### Testing VE assumption

The mean vignette difficulty ratings in the mobility domain increased with increasing severity level of the vignette across all sites (Table 2). This indicated that overall participants understood mobility dysfunction levels described by the vignettes in the same way across sites. This was also seen for the cognition vignettes for all sites except Kenya where the mean rating for learning vignette severity level 4 was lower than that of severity level 3 and in India where the mean ratings for both memory and learning vignette severity level 5 were lower than that for severity level 4 though these differences were not significant.

The assumption of VE was formally tested in 9,375 and 8,788 individuals across the eight sites in the domains of mobility and cognition, respectively. It was seen that the VE assumption was strongly violated across sites both in mobility and cognition domains (Table 3). However, when VE assumption was tested within each site, it was seen that it was not violated in Ghana, Kenya, South Africa, and India for mobility (*p*-value for global test >.05). Individual characteristics which influenced the differential understanding of mobility vignettes were: (i) age in Vietnam; (ii) age and/or education in Tanzania and Indonesia; and (iii) age and/or sex in Bangladesh (Table 4). In the cognition domain, the pattern was less apparent. The assumption of VE was not violated in Kenya, South Africa, Tanzania, and India for the memory vignettes. However, it was violated in Ghana and South Africa and all the Asian sites except India for the learning vignettes which were driven largely by age and education, respectively. The individual characteristics which drove the violation of VE assumption were sex and education in Bangladesh, education in Indonesia, and age in Vietnam for cognition vignettes.

**Table 3:** Wald tests for vignette equivalence between countries for mobility (*N*=9,375) and cognition (*N*=8,788) domain

	Mobility – moving around	Mobility – vigorous activity	Cognition – memory	Cognition – learning
	
	Wald test (*p*-value)	Wald test (*p*-value)	Wald test (*p*-value)	Wald test (*p*-value)
Global test (*df*=28)	1157.9 (.000)	1191.8 (.000)	3039.2 (.000)	2732.6 (.000)
Nairobi, Kenya (*df*=4)	14.1 (.006)	58.5 (.000)	38.6 (.000)	109.2 (.000)
Agincourt, South Africa (*df*=4)	26.7 (.000)	15.8 (.003)	44.0 (.000)	82.8 (.000)
Ifakara, Tanzania (*df*=4)	32.6 (.000)	29.7 (.000)	310.6 (.000)	353.2 (.000)
Matlab, Bangladesh (*df*=4)	44.6 (.000)	44.9 (.000)	106.6 (.000)	107.1 (.000)
Vadu, India (*df*=4)	88.4 (.000)	103.6 (.000)	311.6 (.000)	327.9 (.000)
Purworejo, Indonesia (*df*=4)	46.7 (.000)	37.7 (.000)	74.8 (.000)	105.4 (.000)
Filabavi, Vietnam (*df*=4)	15.5 (.003)	17.4 (.001)	75.2 (.001)	85.7 (.000)

Reference category is Navrongo, Ghana. *p*-Value in parenthesis.

**Table 4:** Wald tests for vignette equivalence within each country for mobility (*N*=9,375) and cognition (*N*=8,788) domain

	Navrongo, Ghana (*p*-value)	Nairobi, Kenya (*p*-value)	Agincourt, South Africa (*p*-value)	Ifakara, Tanzania (*p*-value)	Matlab, Bangladesh (*p*-value)	Vadu, India (*p*-value)	Purworejo, Indonesia (*p*-value)	Filabavi, Vietnam (*p*-value)
Mobility – moving around								
Global test (*df*=24)	29.9 (.186)	19.4 (.731)	17.3 (.831)	47.8 (.002)	79.3 (.000)	19.3 (.735)	62.2 (.000)	130.1 (.000)
Males (*df*=4)	2.3 (.683)	3.9 (.422)	4.3 (.363)	2.4 (.663)	51.9 (.000)	2.6 (.617)	4.5 (.336)	4.3 (.360)
Age (*df*=12)	20.5 (.058)	13.3 (.344)	6.7 (.874)	22.0 (.037)	21.7 (.040)	7.2 (.843)	18.2 (.107)	78.0 (.000)
Education (*df*=8)	11.8 (.157)	2.9 (.939)	5.9 (.652)	16.1 (.040)	6.3 (.608)	7.5 (.480)	21.1 (.006)	6.9 (.539)
Mobility – vigorous activity								
Global test (*df*=24)	27.6 (.275)	27.4 (.284)	14.3 (.937)	50.2 (.001)	69.6 (.000)	19.8 (.706)	66.9 (.000)	93.9 (.000)
Males (*df*=4)	3.4 (.494)	4.3 (.361)	1.4 (.839)	5.0 (.286)	48.9 (.000)	0.2 (.997)	7.4 (.113)	4.9 (.293)
Age (*df*=12)	20.3 (.060)	14.5 (.269)	9.1 (.688)	23.4 (.024)	15.6 (.206)	10.2 (.590)	29.4 (.003)	37.6 (.000)
Education (*df*=8)	4.5 (.805)	9.0 (.342)	5.1 (.738)	14.3 (.074)	7.5 (.482)	9.9 (.267)	12.1 (.143)	9.8 (.272)
Cognition – memory								
Global test (*df*=24)	131.8 (.000)	30.6 (.164)	10.7 (.873)	18.9 (.757)	69.0 (.000)	25.8 (.362)	100.2 (.000)	93.5 (.000)
Males (*df*=4)	2.4 (.662)	4.3 (.362)	8.3 (.081)	3.7 (.437)	40.2 (.000)	1.2 (.867)	5.3 (.254)	3.6 (.454)
Age (*df*=12)	124.8 (.000)	13.6 (.326)	2.8 (.902)	5.3 (.946)	16.8 (.154)	19.3 (.080)	12.7 (.390)	53.6 (.000)
Education (*df*=8)	.7 (.995)	11.4 (.180)	.18 (.999)	7.4 (.493)	18.7 (.016)	5.4 (.705)	54.6 (.000)	7.9 (.434)
Cognition – learning								
Global test (*df*=24)	175.9 (.000)	19.6 (.716)	106.6 (.000)	13.6 (.953)	72.1 (.000)	19.6 (.715)	100.1 (.000)	64.0 (.000)
Males (*df*=4)	3.5 (.480)	2.6 (.624)	8.7 (.068)	3.7 (.439)	37.6 (.000)	4.4 (.353)	2.5 (.636)	1.6 (.800)
Age (*df*=12)	5.4 (.932)	11.2 (.512)	97.9 (.000)	7.1 (.845)	11.5 (.483)	8.6 (.729)	16.4 (.171)	34.4 (.000)
Education (*df*=8)	163.2 (.000)	6.4 (.593)	0.8 (.944)	2.3 (.971)	22.9 (.003)	6.3 (.611)	50.2 (.000)	14.2 (.075)

Reference category is females for sex; age group 50–59 years for age; and primary or less for education.

**Fig. 1:**
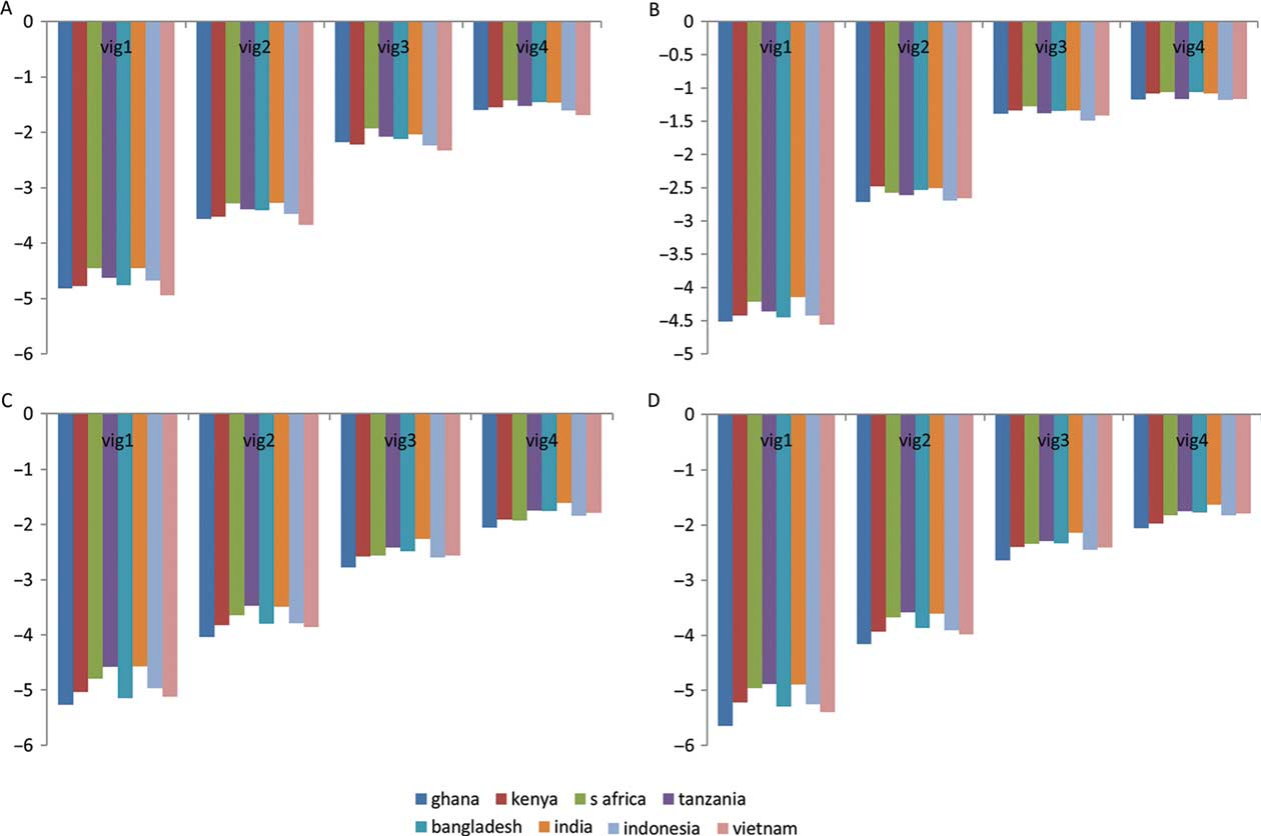
Predicted vignette locations (relative to vignette severity level 5) for mobility (*N*=9,375) and cognition domain (*N*=8,788) identified from HOPIT model 4. Reference category is Navrongo, Ghana: (a) mobility – difficulty in moving around; (b) mobility – difficulty in vigorous activity; (c) cognition – difficulty with memory; (d) cognition – difficulty with learning. *Y*-axis is standardized to SD units of vignette severity level 5 to allow comparison of perceived vignette locations.

However, a less stringent graphical way of testing the assumption of VE showed that there were minimal differences across sites in the predicted locations of each of the mobility vignette (Fig. 1a and b). A consistent increasing trend in predicted location was also seen from vignette severity level 1 to vignette severity level 4 in reference to vignette severity level 5. In contrast, Tanzania and India had lower predicted locations for cognition vignettes compared to the other sites (Fig. 1c and d).

### Testing RC assumption

The assumption of RC was tested in 293 (Navrongo – 148; Agincourt –105; Vadu – 40) and 373 (Navrongo – 151; Agincourt – 110; Vadu – 112) individuals in the mobility and cognition domain, respectively, in the three sites that had administered mobility and cognition tests as part of the fuller version of SAGE. It was seen that the assumption of RC was strongly violated across sites (Table 5) and within sites (Table 6) for both mobility and cognition driven by age, sex, and education.

**Table 5:** Likelihood ratio tests for response consistency between regions for mobility (*N*=293) and cognition (*N*=373) domain

	Mobility – moving around	Mobility – vigorous activity	Cognition – memory	Cognition – learning
	
	LR test (*p*-value)	LR test (*p*-value)	LR test (*p*-value)	LR test (*p*-value)
Global test (*df*=32)	2037.8 (.000)	2053.9 (.000)	2948.4 (.000)	3053.6 (.000)
country (*df*=24)	1764.0 (.000)	2188.0 (.000)	1111.6 (.000)	1166.7 (.000)

**Table 6:** Likelihood ratio tests for response consistency within each country for mobility (*N*=293) and cognition (*N*=373) domain

	Navrongo, Ghana	Agincourt, South Africa	Vadu, India
	
	LR test (*p*-value)	LR test (*p*-value)	LR test (*p*-value)
Mobility – moving around			
Global test (*df*=24)	1010.8 (.000)	191.1 (.000)	312.7 (.000)
Males (*df*=20)	1005.9 (.000)	197.8 (.000)	324.9 (.000)
Age (*df*=12)	1023.7 (.000)	223.3 (.000)	316.9 (.000)
Education (*df*=16)	1011.1 (.000)	190.4 (.000)	311.7 (.000)
Mobility – vigorous activity			
Global test (*df*=24)	1242.2 (.000)	251.5 (.000)	379.5 (.000)
Males (*df*=20)	1241.4 (.000)	256.4 (.000)	391.6 (.000)
Age (*df*=12)	1245.0 (.000)	285.5 (.000)	397.0 (.000)
Education (*df*=16)	1266.9 (.000)	264.4 (.000)	394.6 (.000)
Cognition – memory			
Global test (*df*=24)	1629.8 (.000)	287.6 (.000)	889.1 (.000)
Males (*df*=20)	1627.5 (.000)	293.0 (.000)	893.9 (.000)
Age (*df*=12)	1631.8 (.000)	295.9 (.000)	877.9 (.000)
Education (*df*=16)	1628.4 (.000)	302.8 (.000)	894.2 (.000)
Cognition – learning			
Global test (*df*=24)	1709.5 (.000)	293.8 (.000)	970.5 (.000)
Males (*df*=20)	1701.8 (.000)	292.7 (.000)	971.0 (.000)
Age (*df*=12)	1708.0 (.000)	289.2 (.000)	950.7 (.000)
Education (*df*=16)	1708.2 (.000)	297.0 (.000)	978.3 (.000)

**Fig. 2:**
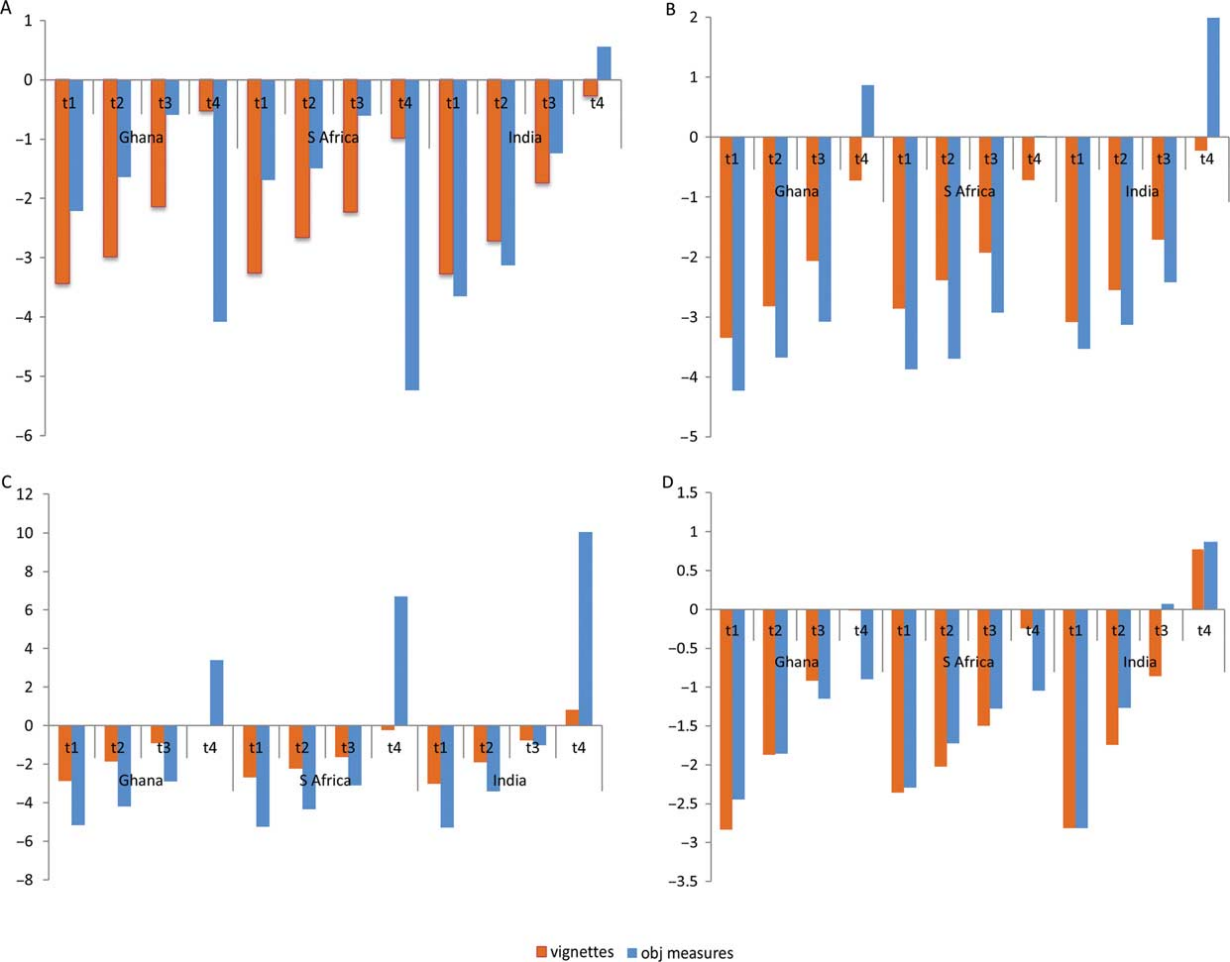
Predicted threshold locations for mobility (*N*=293) and cognition (*N*=373) identified from vignettes (HOPIT model 1) and from objective measures (HOPIT model 2): (a): mobility – difficulty in moving around; (b) mobility – difficulty in vigorous activity; (c) cognition – difficulty with memory; (d) cognition – difficulty with learning. *Y*-axis is standardized to SD units of vignette severity level 5 identified from HOPIT model 1 to allow comparison of perceived threshold locations.

Figure 2 compares the location of predicted thresholds used by the three sites for rating vignettes and for self-rating as derived from objective measures for mobility and cognition. There was a marked difference in the location of the predicted thresholds (test for equality of threshold locations) as identified from both models in all the three sites for both mobility and cognition which suggested that within each site participants used thresholds differently when rating vignettes and self-rating thereby violating the RC assumption. However, when trend lines for the thresholds used for vignette ratings and the thresholds used for self-rating derived from the objective measures model are compared (visual test for equality of distance between thresholds), it was seen that their slopes were moderately similar for moving around and learning domains for India, whereas the regression line slopes were markedly different for Ghana and South Africa. This suggested that the assumption of RC may not be violated for mobility and learning domain in India if a less stringent test (equality of distance between thresholds) was used as compared to the more stringent test of equality of thresholds.

## Discussion

Our study provides evidence of violations of assumptions of response consistence and VE when anchoring vignettes are sought to adjust self-rating responses for RH amongst respondents from eight low- and middle-income countries in Asia and Africa. Evidence from earlier studies, all from Europe or the United States, has been mixed. Some studies have shown violation of these assumptions (27, 28), while others have shown adherence to these assumptions (9, 20, 21). The lack of adherence to assumptions in our study could be because individuals or groups of individuals understood vignettes differently and/or used different thresholds in rating vignettes and their own disability in mobility and cognition. This in turn could be a function of the wording of the anchoring vignette and the rating question, the context in which it was understood, and the level of understanding of the respondent of the five-point ordinal rating scale.

In this article, we analyzed vignettes in two distinct and dissimilar domains of physical and mental health viz. mobility and cognition. We showed that within a country context, older adults (mostly from Africa except Tanzania) understood mobility vignettes in the same way, while in some countries (mostly Asian except India), they understood them differently whereby the variability was driven by the influence of age, sex, and education. This pattern of similar or differential understanding of vignettes by countries was less apparent in the case of cognition vignettes. A less stringent way of testing VE assumption by visual comparison of predicted locations of vignettes suggested that mobility (but cognition less so) vignettes were understood in the same way by older adults from all countries. Finally, there was evidence of violation of the assumption of RC both across countries and within country. However, a less stringent way of visual comparison showed that the RC assumption may not be violated for mobility and cognition vignettes for India. Overall, our study showed a pattern that mobility vignettes are probably better understood by older adults than cognition vignettes.

We evaluated the ‘informativeness’ of each possible set of vignettes by estimating the ‘minimum entropy’ function (results not shown). Both assumptions were still violated even with a smaller subset of vignettes. Collapsing the response categories from five to fewer categories may improve the possibility of the assumptions being met. However, this strategy would be valid if adopted *a priori* as the response category thresholds used by respondents on a four-point ordinal scale may not necessarily be the same as the thresholds derived by collapsing a five-category response to a four-category response *post-priori*. We also chose not to use non-parametric or parametric statistical models which required less strict assumptions (19, 26) to ensure that the assumptions of VE and RC were met.

Our study was limited by the smaller samples available for testing the assumption of RC compared to VE and that this assumption could only be tested in Ghana, South Africa, and India. When we tested RC assumption, that is, compared the model which predicted thresholds used for rating vignettes with the model which derived thresholds based on objective measures to see whether participants used the same thresholds for self-rating and vignette rating, we presumed – justifiably or otherwise – that the objective measures of mobility (normal walk speed, etc.) and cognition (verbal recall, etc.) would capture all the co-variation between the latent mobility and cognition, and the observable characteristics that may influence RH. If so, then any remaining systematic variation seen in self-rating after conditioning on these objective measures could be attributed to RH. We used vignettes adapted from the World Health Survey of 2003, which had been implemented in 70 countries; further research is needed to see if revising the contents and wording of the vignettes (especially for memory and learning function) improves the performance of vignettes both from the perspective of VE as well as RC.

Despite the time and effort, vignettes are important as they provide information on whether individuals or groups of individuals use different thresholds to rate health. Assuming that the health level described by a vignette is understood in the same way by individuals (VE), vignette ratings will identify RH; and assuming that individuals will use the same thresholds to rate vignettes as they rate their own health (RC), vignette ratings will allow the self-rating of their own health to be adjusted for RH. These are essential requirements before any self-rated health function can be compared between individuals or groups of individuals.

## Appendix

**Appendix 1:** Text of mobility and cognition vignettes

Domain question	Vignette
Mobility – overall in the last 30 days, how much difficulty did [you/name_] have …. with moving around? …. with vigorous activity (such as cycling, working in fields, etc.)?	*Severity level 1:* [name_a] has no problems with walking, running or using her hands, arms and legs. She jogs 4 kilometers twice a week. *Severity level 2:* [name_b] is able to walk distances of up to 200 meters without any problems but feels tired after walking 1 kilometer or climbing up more than one flight of stairs. He has no problems with day-to-day physical activities, such as carrying food from the market. *Severity level 3:* [name_c] does not exercise. She cannot climb stairs or do other physical activities because she is obese. She is able to carry the groceries and do some light household work. *Severity level 4:* [name_d] has a lot of swelling in his legs due to his health condition. He has to make an effort to walk around his home as his legs feel heavy. *Severity level 5:* [name_e] is paralyzed from the neck down. He is unable to move his arms and legs or to shift body position. He is confined to bed.
Cognition – Overall in the last 30 days, how much difficulty did [you/name_] have …. with concentrating or remembering things? …. learning a new task (e.g. learning how to get to a new place, learning a new game, learning a new recipe)?	*Severity level 1:* [name_a] is very quick to learn new skills at his work. He can pay attention to the task at hand for long uninterrupted periods of time. He can remember names of people, addresses, phone numbers and such details that go back several years. *Severity level 2:* [name_b] can concentrate while watching TV, reading a magazine or playing a game of cards or chess. He can learn new variations in these games with small effort. Once a week he forgets where his keys or glasses are, but finds them within 5 minutes. *Severity level 3:* [name_c] can find her way around the neighborhood and know where her own belongings are kept but struggles to remember how to get to a place she has only visited once or twice. She is keen to learn new recipes but finds that she often makes mistakes and has to reread several times before she is able to do them properly. *Severity level 4:* [name_d] cannot concentrate for more than 15 minutes and has difficulty paying attention to what is being said to him. Whenever he starts a task, he never manages to finish it and often forgets what he was doing. He is able to learn the names of people he meets but cannot be trusted to follow directions to a store by himself. *Severity level 5:* [name_e] does not recognize even close relatives and gets lost when he leaves the house unaccompanied. Even when prompted, he shows no recollection of events or recognition of relatives. It is impossible for him to acquire any new knowledge as even simple instructions leave him confused.

**Appendix 2:** Summary of ordering of vignettes for mobility (*N*=9,375) and cognition (*N*=8,788) domain. Vignettes 1–5 ordered by increasing level of severity. Upper triangle = *p*_*i*<*j*_−*p*_*j*<*i*_. Lower triangle = 1 − *p*_*i < j*_−*p*_*j < i*_, where *p*_*i*<*j*_ is the proportion of respondents rating vignette *i* < vignette *j* and *p*_*j*<*i*_ is the proportion rating vignette *j* < vignette *i*. Negative values in upper triangle of matrix suggest mis-ordering of corresponding vignette pair (in boldface). Large values in lower triangle suggest high proportion of tied ratings for corresponding vignette pair.

Navrongo, Ghana	Mobility – moving around					Mobility – vigorous activity				
	Vig_1	Vig_2	Vig_3	Vig_4	Vig_5	Vig_1	Vig_2	Vig_3	Vig_4	Vig_5
Vig_1	NA	.57	.91	.93	.93	NA	.81	.95	.94	.93
Vig_2	.34	NA	.71	.87	.93	.13	NA	.71	.78	.89
Vig_3	.04	.09	NA	.28	.80	.03	.19	NA	.11	.55
Vig_4	.15	.48	.02	NA	.69	.21	.59	.05	NA	.50
Vig_5	.02	.02	.12	.19	NA	.02	.02	.34	.37	NA
	Cognition – memory					Cognition – learning				
	Vig_1	Vig_2	Vig_3	Vig_4	Vig_5	Vig_1	Vig_2	Vig_3	Vig_4	Vig_5
Vig_1	NA	.49	.88	.95	.97	NA	.57	.93	.96	.98
Vig_2	.40	NA	.62	.83	.95	.38	NA	.69	.84	.95
Vig_3	.11	.11	NA	.40	.91	.05	.11	NA	.31	.91
Vig_4	.21	.32	.03	NA	.86	.23	.38	.03	NA	.83
Vig_5	.03	.01	.05	.10	NA	.04	.01	.05	.13	NA
Nairobi, Kenya	Mobility – moving around					Mobility – vigorous activity				

	Vig_1	Vig_2	Vig_3	Vig_4	Vig_5	Vig_1	Vig_2	Vig_3	Vig_4	Vig_5
Vig_1	NA	.45	.80	.95	.92	NA	.88	.92	.94	.91
Vig_2	.45	NA	.56	.89	.91	.08	NA	.16	.39	.63
Vig_3	.16	.08	NA	.57	.82	.04	.43	NA	.28	.48
Vig_4	.27	.32	.01	NA	.64	.31	.46	.24	NA	.30
Vig_5	.02	.03	.10	.25	NA	.03	.05	.34	.53	NA
	Cognition – memory					Cognition – learning				
	Vig_1	Vig_2	Vig_3	Vig_4	Vig_5	Vig_1	Vig_2	Vig_3	Vig_4	Vig_5
Vig_1	NA	.54	.90	.88	.96	NA	.23	.85	.72	.94
Vig_2	.33	NA	.66	.70	.95	.57	NA	.74	.57	.89
Vig_3	.05	.20	NA	.18	.73	.04	.19	NA	**−.11**	.68
Vig_4	.22	.26	.03	NA	.60	.11	.34	.70	NA	.71
Vig_5	.04	.02	.25	.30	NA	.21	.02	.25	.22	NA
Agincourt, South Africa	Mobility – moving around					Mobility – vigorous activity				

	Vig_1	Vig_2	Vig_3	Vig_4	Vig_5	Vig_1	Vig_2	Vig_3	Vig_4	Vig_5
Vig_1	NA	.40	.74	.81	.81	NA	.47	.80	.84	.84
Vig_2	.49	NA	.61	.70	.80	.38	NA	.63	.69	.79
Vig_3	.09	.13	NA	.12	.52	.09	.20	NA	.14	.51
Vig_4	.15	.50	.07	NA	.53	.15	.50	.08	NA	.47
Vig_5	.10	.08	.32	.29	NA	.09	.07	.29	.38	NA
	Cognition – memory					Cognition – learning				
	Vig_1	Vig_2	Vig_3	Vig_4	Vig_5	Vig_1	Vig_2	Vig_3	Vig_4	Vig_5
Vig_1	NA	.48	.63	.55	.70	NA	.40	.56	.51	.64
Vig_2	.29	NA	.41	.47	.71	.27	NA	.25	.45	.61
Vig_3	.19	.22	NA	.20	.60	.15	.23	NA	.29	.53
Vig_4	.23	.42	.15	NA	.62	.28	.32	.19	NA	.47
Vig_5	.15	.13	.19	.20	NA	.16	.12	.23	.21	NA
Ifakara, Tanzania	Mobility – moving around					Mobility – vigorous activity				

	Vig_1	Vig_2	Vig_3	Vig_4	Vig_5	Vig_1	Vig_2	Vig_3	Vig_4	Vig_5
Vig_1	NA	.49	.80	.89	.93	NA	.65	.84	.87	.92
Vig_2	.38	NA	.53	.71	.89	.21	NA	.45	.56	.83
Vig_3	.14	.18	NA	.23	.70	.09	.28	NA	.10	.62
Vig_4	.28	.41	.06	NA	.57	.30	.49	.10	NA	.54
Vig_5	.05	.03	.20	.32	NA	.06	.04	.26	.34	NA
	Cognition – memory					Cognition – learning				
	Vig_1	Vig_2	Vig_3	Vig_4	Vig_5	Vig_1	Vig_2	Vig_3	Vig_4	Vig_5
Vig_1	NA	.37	.50	.59	.63	NA	.33	.50	.57	.63
Vig_2	.37	NA	.22	.41	.52	.38	NA	.26	.38	.49
Vig_3	.27	.28	NA	.26	.45	.27	.27	NA	.21	.40
Vig_4	.39	.33	.12	NA	.30	.36	.38	.15	NA	.29
Vig_5	.23	.18	.14	.31	NA	.23	.18	.16	.32	NA
Matlab, Bangladesh	Mobility – moving around					Mobility – vigorous activity				

	Vig_1	Vig_2	Vig_3	Vig_4	Vig_5	Vig_1	Vig_2	Vig_3	Vig_4	Vig_5
Vig_1	NA	.60	.90	.96	.98	NA	.87	.93	.96	.97
Vig_2	.34	NA	.45	.76	.89	.09	NA	.30	.52	.66
Vig_3	.07	.15	NA	.52	.79	.03	.32	NA	.23	.41
Vig_4	.31	.32	.08	NA	.38	.39	.54	.28	NA	.19
Vig_5	.02	.01	.18	.47	NA	.02	.01	.52	.70	NA
	Cognition – memory					Cognition – learning				
	Vig_1	Vig_2	Vig_3	Vig_4	Vig_5	Vig_1	Vig_2	Vig_3	Vig_4	Vig_5
Vig_1	NA	.66	.95	.97	.97	NA	.44	.91	.93	.96
Vig_2	.28	NA	.71	.89	.94	.46	NA	.74	.85	.92
Vig_3	.02	.08	NA	.39	.58	.06	.10	NA	.23	.50
Vig_4	.19	.36	.03	NA	.28	.18	.38	.05	NA	.32
Vig_5	.02	.02	.29	.52	NA	.04	.02	.32	.46	NA
Vadu, India	Mobility – moving around					Mobility – vigorous activity				

	Vig_1	Vig_2	Vig_3	Vig_4	Vig_5	Vig_1	Vig_2	Vig_3	Vig_4	Vig_5
Vig_1	NA	.44	.67	.73	.78	NA	.51	.68	.71	.72
Vig_2	.27	NA	.42	.69	.77	.31	NA	.33	.53	.62
Vig_3	.17	.19	NA	.38	.67	.15	.21	NA	.31	.54
Vig_4	.32	.33	.10	NA	.58	.29	.33	.14	NA	.44
Vig_5	.12	.10	.18	.20	NA	.12	.11	.22	.28	NA
	Cognition – memory					Cognition – learning				
	Vig_1	Vig_2	Vig_3	Vig_4	Vig_5	Vig_1	Vig_2	Vig_3	Vig_4	Vig_5
Vig_1	NA	.28	.67	.75	.65	NA	.28	.72	.72	.65
Vig_2	.42	NA	.58	.65	.55	.44	NA	.63	.63	.56
Vig_3	.12	.18	NA	.20	.08	.13	.17	NA	.10	.06
Vig_4	.21	.56	.16	NA	**−.11**	.19	.48	.20	NA	**−.08**
Vig_5	.15	.18	.53	.53	NA	.14	.17	.49	.54	NA
Purworejo, Indonesia	Mobility – moving around					Mobility – vigorous activity				

	Vig_1	Vig_2	Vig_3	Vig_4	Vig_5	Vig_1	Vig_2	Vig_3	Vig_4	Vig_5
Vig_1	NA	.47	.69	.86	.95	NA	.66	.81	.91	.95
Vig_2	.39	NA	.38	.72	.92	.22	NA	.40	.70	.88
Vig_3	.22	.20	NA	.45	.84	.11	.22	NA	.39	.76
Vig_4	.38	.36	.05	NA	.65	.35	.42	.08	NA	.56
Vig_5	.09	.02	.12	.29	NA	.04	.02	.19	.36	NA
	Cognition – memory					Cognition – learning				
	Vig_1	Vig_2	Vig_3	Vig_4	Vig_5	Vig_1	Vig_2	Vig_3	Vig_4	Vig_5
Vig_1	NA	.45	.80	.90	.94	NA	.37	.83	.89	.93
Vig_2	.44	NA	.53	.78	.89	.50	NA	.62	.79	.89
Vig_3	.12	.14	NA	.47	.75	.10	.13	NA	.43	.72
Vig_4	.29	.34	.07	NA	.45	.22	.37	.07	NA	.43
Vig_5	.06	.03	.18	.41	NA	.06	.04	.21	.43	NA
Filabavi, Vietnam	Mobility – moving around					Mobility – vigorous activity				

	Vig_1	Vig_2	Vig_3	Vig_4	Vig_5	Vig_1	Vig_2	Vig_3	Vig_4	Vig_5
Vig_1	NA	.40	.82	.96	.98	NA	.88	.94	.96	.97
Vig_2	.55	NA	.59	.84	.96	.08	NA	.51	.65	.83
Vig_3	.14	.10	NA	.45	.89	.03	.29	NA	.17	.51
Vig_4	.25	.41	.02	NA	.77	.35	.61	.15	NA	.36
Vig_5	.02	.00	.10	.20	NA	.02	.01	.45	.59	NA
	Cognition – memory					Cognition – learning				
	Vig_1	Vig_2	Vig_3	Vig_4	Vig_5	Vig_1	Vig_2	Vig_3	Vig_4	Vig_5
Vig_1	NA	.54	.90	.94	.94	NA	.43	.93	.93	.95
Vig_2	.39	NA	.71	.88	.91	.48	NA	.79	.88	.92
Vig_3	.06	.08	NA	.46	.67	.03	.07	NA	.41	.65
Vig_4	.18	.40	.05	NA	.34	.13	.40	.04	NA	.33
Vig_5	.02	.02	.26	.51	NA	.03	.02	.27	.53	NA
